# Reliability and structural validity of the Norwegian version of the TeamSTEPPS Teamwork Attitudes Questionnaire: A cross‐sectional study among Bachelor of Nursing students

**DOI:** 10.1002/nop2.671

**Published:** 2020-11-04

**Authors:** Tore Karlsen, Marie Louise Hall‐Lord, Sigrid Wangensteen, Randi Ballangrud

**Affiliations:** ^1^ Department of Health Science Gjøvik Faculty of Medicine and Health Sciences Norwegian University of Science and Technology Gjoevik Norway; ^2^ Department of Health Sciences Faculty of Health, Science and Technology Karlstad University Karlstad Sweden

**Keywords:** Norway, nurses, nursing, nursing students, students, teamwork

## Abstract

**Aim:**

To test the reliability and structural validity of the Norwegian version of the TeamSTEPPS^®^ Teamwork Attitudes Questionnaire (T‐TAQ) among Bachelor of Nursing students.

**Design:**

Cross‐sectional study.

**Methods:**

Bachelor of Nursing students (*N* = 1,624) at three campuses in different regions of Norway were invited to complete the survey. The data were analysed with descriptive statistics, Cronbach's alpha and confirmatory factor analysis (CFA). Three models were tested. Model 3 was a post hoc modification with a correlation between four negatively worded items. The data was collected in September 2018 and May‐June 2019.

**Results:**

A total of 509 students were included in the study. Cronbach's alpha ranged from 0.44–0.70 for the dimensions and was 0.79 for the total questionnaire. The fit indexes of model 3 were as follows: RMSEA = 0.043, chi‐square = 724.3 (*p* < .000), normed chi‐square = 1.862, TLI = 0.812 and CFI = 0.832. The questionnaire shows some potential to display attitudes towards teamwork in health care among Bachelor of Nursing students. Low Cronbach's alpha in the dimensions might indicate that the questionnaire should be considered used as a unidimensional questionnaire.

## 1 INTRODUCTION

There is a consensus that teamwork constitutes one of the critical elements in today's highly complex system of delivering safe and effective patient care (Neuhaus et al., 2019; Rosen et al., 2018; Schmutz & Manser, 2013). According to the Institute of Medicine (IOM) report entitled “Health Professions Education: A Bridge to Quality,” teamwork is one of the skills necessary to ensure quality and safety in health care (Knebel & Greiner, 2003). It is therefore vital to incorporate teamwork into the education of healthcare professionals (Dow et al., 2013; Norwegian Ministry of Health & Care Services, 2019; Sherwood & Barsteiner, 2017). Team training has, to a limited extent, been implemented in the education of Bachelor of Nursing students in Norway (Aase et al., 2013). This study is part of a project that aims to create new knowledge regarding the integration of a team training programme into a Norwegian Bachelor of Nursing programme. Changes in attitudes are a frequently used measure of learning outcomes in team training (LaMothe et al., 2016; Reeves et al., 2016; Sweigart et al., 2016; Vertino, 2014); thus, high validity and reliability are essential for questionnaires measuring changes in attitudes (Polit & Yang, 2016).

## 2 BACKGROUND

Team Strategies and Tools to Enhance Performance and Patient Safety (TeamSTEPPS^®^) is a team training programme based on more than 20 years of research examining elements that are essential for providing effective and safe care in health care, including the principles of sustainable implementation (King et al., 2008; Salas et al., 2018). The Agency for Healthcare Research and Quality (AHRQ) developed the TeamSTEPPS^®^ team training programme in cooperation with the Department of Defense (DoD) in the United States (AHRQ, 2012). The programme emphasizes the importance of team structure and four team skills: leadership, situation monitoring, mutual support and communication. The training programme consists of lectures, reinforcement in simulation‐based scenarios, low‐fidelity training and roleplay, feedback and reflection in clinical settings (AHRQ, 2012; Chen et al., 2019). The TeamSTEPPS^®^ team training programme has been used in various healthcare educational settings, such as in nursing education (Gaston, 2018; Goliat et al., 2013; Maguire et al., 2015; Robinson et al., 2018) and in interprofessional educational settings (Chen et al., 2019; Welsch et al., 2018). Previous research has shown positive outcomes of the TeamSTEPPS^®^ team training programme, including reduced patient complications, mortality (Forse et al., 2011) and risk of fall (Spiva et al., 2014). Positive organizational outcomes include an increase in effective patient treatment (Capella et al., 2010) and improved patient safety culture (Aaberg et al., 2019). Learning outcomes show a positive change among students (Maguire et al., 2015; Sweigart et al., 2016) and among healthcare professionals' (Vertino, 2014; Wadsworth, 2019) attitudes towards teamwork after the implementation of TeamSTEPPS^®^. Participants also seem to enjoy attending the team training programme (Thomas & Galla, 2013; Welsch et al., 2018). These outcomes motivated the research team to design a study to implement TeamSTEPPS^®^ in Bachelor of Nursing education. To our knowledge, no Bachelor of Nursing programme in Europe has implemented the TeamSTEPPS^®^ team training programme.

Methods used to measure attitudes can provide useful information regarding the perception of teamwork behaviour (Frager, 2014; Manser, 2009). According to Ajzen (1991), intentions to perform behaviours can be predicted by attitudes towards the behaviour, subjective norms and perceived behavioural control. Behavioural purposes account for considerable variance in actual practice (Ajzen, 1991). The content of the T‐TAQ was developed based on extensive research on essential teamwork attributes (Baker et al., 2008). According to Baker et al. (2010), the TeamSTEPPS^®^ Teamwork Attitudes Questionnaire (T‐TAQ) was designed to measure attitudes towards the core components of teamwork aligned with the TeamSTEPPS^®^ team training programme. Data from the questionnaire can be used to assess changes in participants' attitudes towards teamwork as a result of training, as attitudes are an aspect of learning. The questionnaire may also support quality improvement activities associated with teamwork (Baker et al., 2010). The T‐TAQ is the most frequently used instrument to measure changes in attitude following intervention with the TeamSTEPPS programme in interprofessional education settings (Welsch et al., 2018). The Norwegian version of the T‐TAQ has been validated in a population of healthcare professionals (Ballangrud et al., 2019).

Previous studies have used the T‐TAQ questionnaire to evaluate team training with interprofessional students (Chen et al., 2019; Welsch et al., 2018), nursing students (Gaston, 2018; Godin et al., 2017; LaMothe et al., 2016; Maguire et al., 2015) and healthcare professionals (Grapensteter, 2017; Vertino, 2014). Bachelor's students are a different population from experienced healthcare professionals with respect to knowledge, teamwork and healthcare experience. Therefore, it was essential to validate the questionnaire among Bachelor of Nursing students, as they were the population of interest in this project. According to Wooding et al. (2019), questionnaires should not be reused without consideration of the population studied. Structural validity should be reassessed to obtain valid and reliable results in a new target population (Polit & Yang, 2016). Previous T‐TAQ studies in nursing education have been conducted with relatively small samples (*N* = 7–182) (Gaston, 2018; Goliat et al., 2013; LaMothe et al., 2016; Maguire et al., 2015), which makes it challenging to conduct powerful studies of the validity and reliability of a questionnaire (Polit & Yang, 2016). At this point, we have not found any studies examining the reliability and validity of the T‐TAQ within a population of Bachelor of Nursing students.

### 2.1 Aim of the study

This study aimed to test the reliability and structural validity of the Norwegian version of the T‐TAQ among Bachelor of Nursing students.

## 3 THE STUDY

### 3.1 Design

The study used a cross‐sectional design (Polit & Beck, 2016).

### 3.2 Method

#### 3.2.1 Setting and sample

The study was conducted at a Norwegian university, which offers a Bachelor of Nursing programme at three campuses in three different regions. All students (*N* = 1,624) were invited to participate; 408 were first‐year students, 532 were second‐year students and 684 were third‐year students. According to Polit and Yang (2016), an estimated minimum sample size of ten individuals per item on the questionnaire is necessary for confirmatory factor analysis (CFA), but a larger sample is desirable.

#### 3.2.2 The questionnaire

The T‐TAQ was designed to evaluate the TeamSTEPPS^®^ team training programme (AHRQ, 2014). The T‐TAQ evaluates five dimensions of teamwork: team structure (TS), leadership (L), situation monitoring (S), mutual support (MS) and communication (C). The questionnaire comprises 30 items, with six items in each dimension. Four items are negatively worded (MS20, MS21, MS24 and C30) (Table 2). The questionnaire was cross‐culturally translated as recommended (c.f. Brislin, 1970), and some semantic and conceptual changes were made after a pilot test. The analysis showed Cronbach's alpha values from 0.53–0.76, a normed chi‐square of 1.896, an RMSEA of 0.061, a TLI of 0.773 and a CFI of 0.794 (Ballangrud et al., 2019). The respondents score each item on a five‐point Likert scale to indicate their level of agreement from strongly disagree (1) to strongly agree (5) with the statement. Central teamwork constructs were explained on the first page of the questionnaire. The students were asked to complete background data on sex, age, study progression, campus, former higher education and work experience in health care.

#### 3.2.3 Face validity

We invited a convenience sample of final‐year Bachelor of Nursing students (*N* = 40) who did not participate in the main study to take part in an email pilot survey to evaluate the face validity of the T‐TAQ. The students were asked to respond to each item, as well as to answer additional questions about to what extent they perceived the items as clear and understandable, as well as how easy it was to choose an option on the Likert scale. The respondents had the opportunity to comment with suggestions on how to improve the questionnaire. Based on the response (*N* = 10), we added supplementary information to items 13 and 14.

#### 3.2.4 Data collection

The data collection took place in September 2018 and May–June 2019. A paper version of the T‐TAQ (paper survey) was distributed to first‐year students (*N* = 408) who were present during a class. The survey took place after their first clinical placement. The students who wanted to participate answered the survey and returned the questionnaire as they left the class.

Because second‐ and third‐year students in clinical placements were spread over a large geographic area, an electronic survey was administered as an email survey to these students (*N* = 1,216). For the students who accepted the invitation, a hyperlink directed them to the questionnaire. Reminders were sent after 3 and 7 days.

#### 3.2.5 Analysis

The statistical software IBM SPSS version 26 (2019) and SPSS AMOS version 25 were used to analyse the data. Before the analysis, the scores of the four negatively worded items were reversed. Descriptive statistics were used to analyse the background data, teamwork dimensions and items. Cronbach's alpha was used to calculate internal consistency; a value above 0.70 was considered acceptable (Polit & Yang, 2016; Tavakol & Dennick, 2011).

We examined the data for missing item responses before the CFA analysis. The analysis of missing data resulted in a listwise deletion of 32 respondents before the CFA was conducted with a sample of 477. A rule of thumb is a sample size of at least 10 individuals per item for the analysis (Polit & Yang, 2016).

A CFA makes it possible to test how well each item measures the dimension that it is supposed to measure and whether the items explain the variance in the latent dimensions (Brown & Moore, 2012). The structure of the Norwegian version of the questionnaire is based on the original instrument developed by Baker et al. (2010) and hypothesizes that the variance in the responses to the items reflects the variance in the latent dimensions on which the manifest items are loaded (Brown, 2006; Polit & Yang, 2016). The regression coefficient between the first variable and the latent construct in each dimension was fixed to 1, and the unstandardized regression coefficients from the error terms to the measured variables were also fixed to 1 (Polit & Yang, 2016). The error (e) variance for each item indicates the reliability of the observed variables and is influenced by the random measurement error (Byrne, 2010).

We tested the goodness‐of‐fit of three models. Model 1 was based on the unmodified T‐TAQ questionnaire structure and Model 2 tested the same model with the sample randomly split in half to examine the stability of the results in Model 1 (Schreiber et al., 2006). Model 3 calculated the model fit with a post hoc modification. We wanted to test whether an intercorrelation between error variances among the four negatively worded items (MS20, MS21, MS24 and C30) could result in a better model fit. This was based on poor factor loading and a hypothesis of intercorrelation based on the shared reversion of the items.

The model fit was estimated with equations of four recommended fit indexes in all three models (Polit & Yang, 2016; Schreiber et al., 2006). Absolute fit indexes indicate how well the T‐TAQ model fitted the data and were calculated with the chi‐square, normed chi‐square and root mean square error of approximation (RMSEA). The chi‐square statistic should be nonsignificant with a *p*‐value > .05. The normed chi‐square (*χ* ^2^/*df*) should be <2, and the RMSEA has a threshold value of ≤0.06 (Hu & Bentler, 1999; Polit & Yang, 2016). Comparative fit indexes compare the model with a null model where all of the variables are uncorrelated (Polit & Yang, 2016). These indexes were calculated with the comparative fit index (CFI) and the Tucker–Lewis fit index (TLI). The CFI and TLI should have values close to 1.0, and threshold values are ≥0.95 (Hu & Bentler, 1999; Polit & Yang, 2016).

As a part of the CFA, correlations between the latent dimensions were analysed. Since all dimensions address aspects of teamwork, a positive correlation between the latent dimensions was hypothesized (Polit & Yang, 2016).

#### 3.2.6 Ethics

The study was conducted according to the Helsinki Declaration for ethical principles of research (WMA, 2013). The study was approved by the Norwegian Social Science Data Service (NSD ID: 738592) and by the university involved. The invited students obtained written information about the aim of the study and were informed that responding to the questionnaire was voluntary and had no consequences for their educational progression. Returning the questionnaire was considered to indicate consent to participate in the study.

## 4 RESULTS

A total of 509 students answered the questionnaire (31.3%). The email survey had a response rate of 15.3% and the paper survey had a response rate of 76.2%. The sample characteristics are displayed in Table 1. In short, 61.1% of the respondents were first‐year students, 84.1% were female, the median age was 22 years with a range from 18–55 years and 75.2% had work experience in health care.

**TABLE 1:** Characteristics of the sample (*N* = 509)

Variable	Category	*N*	%
Study progression	First‐year students	311	61.1
	Second‐year students	94	18.5
	Third‐year students	104	20.4
Age median(range)		22 (18–55)	
Sex	Female	428	84.1
	Male	75	14.7
	Missing	6	1.2
Former working experience in healthcare	0 year	110	21.6
	<1 year	74	14.5
	1–2 years	132	25.9
	3–5 years	132	25.9
	>6 years	42	8.3
	Missing	19	3.7

Table 2 shows the mean scores and the standard deviations of the T‐TAQ total scale, the five dimensions and the individual items. The mean score of the items ranged from 3.69 (TS4) to 4.80 (L7). The standard deviation (*SD*) varied between 0.44 (L7) and 1.06 (M20†).

**TABLE 2:** Mean score and standard deviation for T‐TAQ items and dimensions (*N* = 509)

	Items description	Mean	*SD*
	**Team structure (TS)**	**4.17**	**0.38**
TS1	It is important to ask patients and their families for feedback regarding patients' care	4.49	0.63
TS2	Patients are critical component of the care team	4.76	0.50
TS3	The facility's administration influences the success of direct care teams	4.13	0.73
TS4	A team's mission is of greater value than the goals of individual team members	3.69	0.89
TS5	Effective team members can anticipate the needs of other team members	4.12	0.74
TS6	High performing teams in health care share common characteristics with high performing teams in other industries	3.80	0.79
	**Leadership (L)**	**4.46**	**0.39**
L7	It is important for leaders to share information with team members	4.80	0.44
L8	Leaders should create informal opportunities for team members to share information	4.14	0.83
L9	Effective leaders view honest mistakes as meaningful learning opportunities	4.35	0.68
L10	It is a leader's responsibility to model appropriate team behaviour	4.56	0.58
L11	It is important for leaders to take time to discuss with their team members plans for each patient	4.45	0.68
L12	Team leaders should ensure that team members help each other out when necessary	4.46	0.65
	**Situation Monitoring (S)**	**4.22**	**0.52**
S13	Individuals can be taught how to scan the environment for important situational cues	4.24	0.78
S14	Monitoring patients provides an important contribution to effective team performance	4.13	0.93
S15	Even individuals who are not part of the direct care team should be encouraged to scan for and report changes in patient status	4.02	0.93
S16	It is important to monitor the emotional and physical status of other team members	4.16	0.67
S17	It is appropriate for one team member to offer assistance to another who may be too tired or stressed to perform a task	4.45	0.61
S18	Team members who monitor their emotional and physical status on the job are more effective	4.25	0.74
	**Mutual support (MS)**	**4.21**	**0.41**
MS19	To be effective. team members should understand the work of their fellow team members	4.15	0.68
MS20†	Asking for assistance from a team member is a sign that an individual does not know how to do his/her job effectively	3.94	1.06
MS21†	Providing assistance to team members is a sign that an individual does not have enough work to do	4.25	0.72
MS22	Offering to help a fellow team member with his/er individual work tasks is an effective tool for improving team performance	4.28	0.65
MS23	It is appropriate to continue to assert a patient safety concern until you are certain that it has been heard	4.63	0.52
MS24†	Personal conflicts between team members do not affect patient safety	4.01	0.98
	**Communication (C)**	**4.28**	**0.38**
C25	Team that do not communicate effectively significantly increase their risk of committing errors	4.72	0.54
C26	Poor communication is the most common cause of reported errors	4.02	0.73
C27	Adverse events may be reduced by maintaining an information exchange with patients and their families	4.34	0.58
C28	I prefer to work with team members who ask questions about information I provide	3.95	0.74
C29	It is important to have a standardized method for sharing information when handing off patients (e.g. shift exchange. transfer to other units)	4.48	0.60
C30†	It is nearly impossible to train individuals how to be better communicators	4.14	0.84
	T‐TAQ total score	4.27	0.27

Abbreviations: T‐TAQ, TeamSTEPPS Teamwork Attitudes Questionnaire. ^†^Reversed items; Scale: 1, strongly disagree; 2, disagree; 3, neutral; 4, agree; 5, strongly agree.

Cronbach's alpha coefficient for the total questionnaire was 0.79, and the coefficients for each dimension varied from 0.44–0.70, as shown in Table 3. Table 4 shows the fit indexes for the three models.

**TABLE 3:** Cronbach's alpha of T‐TAQ, in the current study and previous studies

Dimensions	*N* of items	Current study (*N* = 509)	Baker et al. (2010) (*N* = 449)	Ballangrud et al. (2019) (*N* = 249)	Brock et al. (2013) (*N* = 149)	Sweigart et al. (2016) (*N* = 109)
Total scale	30	0.79	n/a	0.83	0.93	n/a
Team structure	6	0.46	0.70	0.57	†	0.71
Leadership	6	0.62	0.81	0.76	†	0.82
Situation monitoring	6	0.70	0.83	0.75	†	0.89
Mutual support	6	0.44	0.70	0.53	0.62	0.75
Communication	6	0.56	0.74	0.57	†	0.57

Abbreviation: n/a, not available.

^†^Brock et al. (2013) reported Cronbach's Alpha values of the other dimensions as a range from 0.85–094.

**TABLE 4:** Confirmatory factor analysis (CFA) fit indexes

	CFA index standard	Model 1	Model 2		Model 3
		Total sample without missing (*n* = 477)	Random split half (*n* = 238)	Random split half (*n* = 239)	Correlation between four reversed items (*n* = 477)
Chi‐square		884.2	665.3	629.7	724.3
*p*‐value	>.05	.000	.000	.000	.000
*df*		395	395	395	389
Normed chi‐square	<2	2.239	1.684	1.594	1.862
RMSEA (CI)	≤0.06	0.051 (0.047–0.056)	0.054 (0.047–0.061)	0.050 (0.043–0.057)	0.043 (0.038–0.047)
TLI	>0.95	0.730	0.710	0.743	0.812
CFI	>0.95	0.755	0.737	0.767	0.832

Abbreviations: CFI, Comparative Fit Index; CI, Confidence Interval; df, degree of freedom; RMSEA, Root Mean Squire Error of Approximation; TLI, Tucker‐Lewis Index.

**FIGURE 1:**
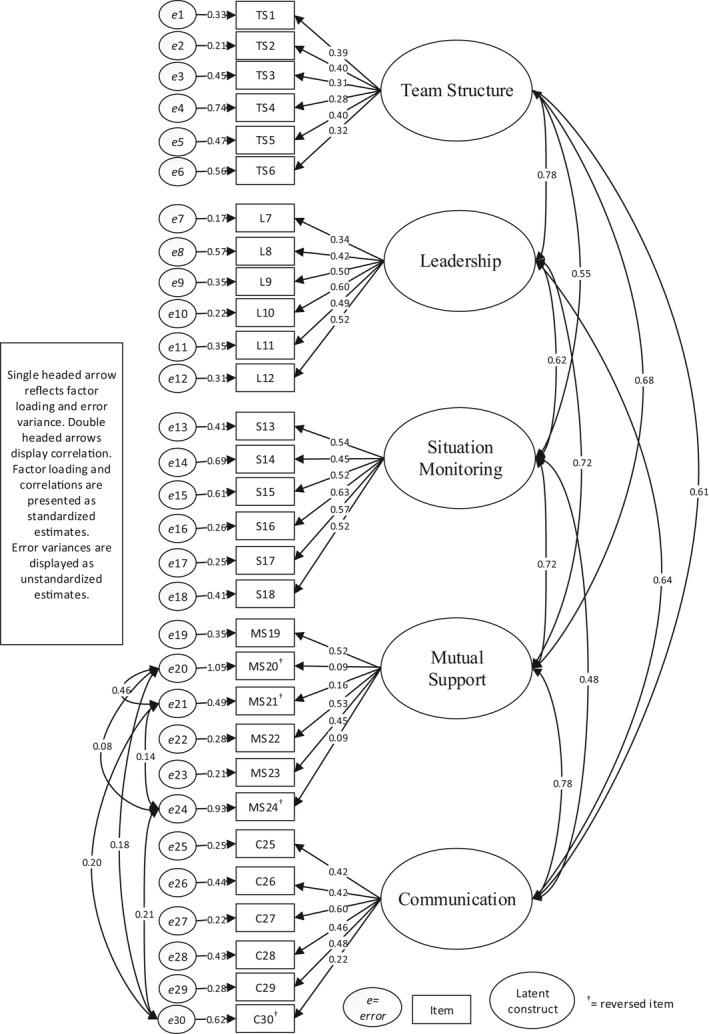
Structural model with factor loading, error variance and correlations

Model 1 had a significant chi‐square value. The normed chi‐square was 2.24. The RMSEA was 0.051, and the TLI and CFI were lower than the threshold values. Model 2 confirmed the stability of the equations in model 1. Model 3 generated the fit indexes after a post hoc modification with the estimation of intercorrelation between error variances (residuals) of the four negatively worded items MS20, MS21, MS24 and C30. Model 3 showed a significant chi‐square value. The normed chi‐square was <2 and the RMSEA was 0.043. The TLI and CFI increased because of model modification but were still lower than the threshold values. The factor loadings, error variances and correlations between dimensions and between the selected error variances in model 3 are displayed in Figure 1.

Standardized factor loading ranged from 0.09–0.63. Of the 30 items, 25 had a factor loading >0.30 to the targeted latent dimension. Situation monitoring shows the highest factor loading for all six items, with a regression coefficient of 0.45–0.63. The mutual support dimension showed the lowest factor loading for two negatively worded items with a value of 0.09 and one item with a value of 0.16. The error variance (e) for all items varied from 0.17–1.05. Model 3 showed positive correlations between the error variances of all the negatively worded items and the highest correlation was between e20–e21. The correlation between dimensions ranged from 0.48–0.78, as shown in Figure 1.

## 5 DISCUSSION

This study aimed to test the reliability and structural validity of the Norwegian version of the T‐TAQ among Bachelor of Nursing students. Cronbach's alpha indicated that the reliability of the total questionnaire was acceptable, although Cronbach's alpha within dimensions ranged from 0.44–0.70. The analysis of goodness‐of‐fit indexes showed acceptable values in two absolute fit indexes (RMSEA, normed chi‐square) and below‐threshold values for the comparative fit indexes (CFI, TLI) and the chi‐square index.

### 5.1 Reliability

The total questionnaire showed acceptable internal consistency with Cronbach's alpha value of 0.79. The questionnaire has 30 items, and Cronbach's alpha value tends to increase with higher number of items (Tavakol & Dennick, 2011). The situation monitoring dimension had a Cronbach's alpha value of 0.70 and indicated acceptable internal consistency. This dimension showed the highest value of internal consistency in both the current study and in previous T‐TAQ studies, as shown in Table 3 (Baker et al., 2008; Ballangrud et al., 2019; Sweigart et al., 2016). Cronbach's alpha value of 0.44 in the mutual support dimension indicated low internal consistency. The low Cronbach's alpha value is congruent with previous research that shows that the mutual support dimension had the lowest reliability of the five dimensions when used in professional healthcare samples (Baker et al., 2010; Ballangrud et al., 2019) as well as in a sample of interprofessional students (Brock et al., 2013). Cronbach's alpha values indicate inter‐item homogeneity (Cronbach, 1951), and a low value may thereby indicate that all the items do not reflect the same latent dimension. Our study showed Cronbach's alpha value of 0.56 in the communication dimension, which is close to the value of 0.57 reported in two previous studies (Ballangrud et al., 2019; Sweigart et al., 2016). A low factor loading of items to the dimensions may partly explain the low values of Cronbach's alpha.

### 5.2 Validity

The RMSEA values were acceptable and indicated a good fit, as the values were below the threshold value and had narrow confidence intervals (Byrne, 2010). This index is considered one of the most informative fit indexes and is widely used to measure how well the correlations of the theoretical model match the observed correlations (Byrne, 2010; Meyers et al., 2016). The RMSEA may be vulnerable with a small sample size (Hu & Bentler, 1999), but the sample size in this study (*N* = 477) is considered acceptable to calculate a valid RMSEA. The number of participants needed is not an exact rule, but ten individuals per estimated item seems to be the consensus (Polit & Yang, 2016; Schreiber et al., 2006). The sample size in our study was equivalent to 70% of the typical sample size in structural equation modelling (*SEM*) studies in nursing research (Sharif et al., 2018).

A perfect fit for a model would be indicated by a nonsignificant chi‐square value (Polit & Yang, 2016). However, for most empirical *SEM* studies, this has been proven to be unrealistic (Byrne, 2010). The chi‐square test is highly sensitive to sample size, a high correlation between the dimensions in the questionnaire and error variance in the model (Kline, 2011). Thus, other fit indexes often receive more attention (Mishra, 2016; Polit & Yang, 2016).

We considered the normed chi‐square acceptable with a value <3 in all three models. There is no consensus regarding whether the cut‐off value should be below 2 or 3 (Polit & Yang, 2016; Schreiber et al., 2006). The normed chi‐square in our study was <2 in two out of three models. The goodness‐of‐fit indexes showed better values from model 1 to model 3 (Polit & Yang, 2016; Schreiber et al., 2006). The comparative fit indexes (TLI and CFI) are below‐threshold values but are, to some degree, considered too strict, especially with complex models (Marsh et al., 2004). The CFI compares the targeted model with a model that has no correlation between the variables, which is unlikely in most models (Rigdon, 1996). Rigdon (1996) claims that the CFI is more suited for explorative factor analyses and small samples and the RMSEA is more suited for more confirmatory, large‐sample cases, as in our study. Absolute fit indexes and comparative fit indexes represent the data from different perspectives and a model with inconsistency may be neither “good” nor “bad” but may have limitations and the results must be interpreted with this in mind (Lai & Green, 2016).

The structural validity of a model demonstrates whether the model measures what it is described to measure and is indicated by the factor loading and associated error variances (Byrne, 2010). Twenty‐five out of 30 items loaded on the targeted latent dimensions with a factor loading above 0.30, which should be considered acceptable, according to Kääriäinen et al. (2011). Situation monitoring shows a factor loading for all items >0.40 and reveals the highest internal consistency. The mutual support dimension has three items with acceptable factor loading and three with low factor loading and shows a low Cronbach's alpha. Negatively worded items loading on the mutual support and communication dimensions may explain why not all fit indexes are within threshold values in this model (Fan & Sivo, 2005). The items with low factor loadings showed similarly high error variances, which indicates that there is a bias that is not a result of variation in the respondents' attitudes towards the targeted dimension. A model should have an appropriate factor loading of items to the latent dimension to be a valid instrument (Byrne, 2010).

According to Mishra (2016), some plausible explanations of error variances might be that respondents have limited experience with the construct, the respondents might not have understood the meaning of the items, or they respond according to social desirability. Cote and Buckley (1987) claim that abstract constructs may be more challenging to measure than concrete constructs are and measurement error in social science research within the education discipline accounts for 30.5% of the variance. We conducted our study in the context of education and measured an abstract construct; thus, variance as a result of measurement error may be plausible.

Model 3 (after post hoc modification) shows that a correlation between error variances of the reversed items strengthens the fit indexes of the model. This confirms that there is a substantial correlation between the error variances for item MS20 and item MS21. These items pertain to seeking and offering assistance and are some of the core elements of mutual support in teamwork (King et al., 2008); furthermore, these two items have both low factor loading and high error variance and the error variance is correlated.

Negatively worded items have both advantages and disadvantages (Polit & Yang, 2016; Weijters & Baumgartner, 2012). Negatively worded items may correct for agreement bias, mainly if the scale comprises equal numbers of regular and negatively worded items (Baumgartner & Steenkamp, 2001). However, it may affect the reliability, goodness‐of‐fit and factor loading of questionnaires (Baumgartner & Steenkamp, 2001). A problem in the T‐TAQ was that the negatively worded items were not balanced through the questionnaire, as all the negatively worded items were in the last two‐thirds of the questionnaire. This location may make the respondents more relaxed and more careless in interpreting and responding to the items (Baumgartner & Steenkamp, 2001).

Baker et al. (2008, p. 7) state in their T‐TAQ manual that “items on the T‐TAQ should not be modified.” The modification of a model should be theoretically justified (Polit & Yang, 2016) as well, and the T‐TAQ is built on a thorough theoretical base (Baker et al., 2010). Our results indicate that the reversed items are troublesome for factor loading and affect the reliability of the dimensions.

Our study shows intercorrelation between dimensions between 0.48–0.78 (Figure 1). The strongest intercorrelation is 0.78 between mutual support and communication and between team structure and leadership. Previous studies of the T‐TAQ show weaker intercorrelation between the latent dimensions (Baker et al., 2010; Ballangrud et al., 2019), which may be attributable to different methods of analysis. The mean score is high in all dimensions, and this is congruent with what the developers of the instrument found (Baker et al., 2010) and what Ballangrud et al. (2019) showed in the Norwegian version. However, several studies show statistical significant changes in pre‐ and post‐test studies used in educational settings (Brock et al., 2013; Goliat et al., 2013; Maguire et al., 2015). This might indicate that the questionnaire is suitable for measuring a change in attitudes among healthcare students.

### 5.3 Limitations

A limitation of this study is that more than 60% of the sample comprised first‐year students. First‐year students are supposed to be both the youngest and the least experienced segment of the sample with respect to teamwork experience and professional knowledge. Another limitation is the use of two different methods of data collection. The email survey invited most of the available students but resulted in a response rate of only 15.3%. It is a known challenge to researchers that email surveys may have lower response rates than other survey methods (Manfreda et al., 2008). Regarding data collection by pen and paper, the number of students responding was limited to the students present in the class. On the other hand, the range and median age and sex of the respondents seem to be representative of the target population in Norway (Statistics, 2018).

## 6 CONCLUSION

The questionnaire shows acceptable absolute fit indexes. The CFA analysis shows acceptable values of RMSEA and normed chi‐square values. Cronbach's alpha coefficient for the total questionnaire was acceptable. However, the internal consistency of four out of five dimensions was low. This study shows that the negatively worded items are troublesome for factor loading and affect the reliability of the dimensions. These results might indicate that the questionnaire should be considered unidimensional when used with undergraduate healthcare students, even if it comprises different fractions of the concept of teamwork. When the questionnaire is applied in educational settings, awareness of some negatively worded items should be highlighted to avoid measurement errors. Further studies are recommended to test the psychometric properties of the T‐TAQ among other Bachelor of Nursing students and among multi‐professional students.

## CONFLICT OF INTEREST

All authors declare no conflict of interest.

## AUTHOR CONTRIBUTION

TK, MH, SW, RB: responsible for the conception and study design. TK: performed the data collection. TK, MH, SW, RB: contributed to the analysis of the data. TK, MH, SW, RB: involved in drafting the manuscript and revising it critically for important intellectual content. All authors have read and approved the final manuscript.

## Supporting information

Supplementary Information

## Data Availability

The data that support the findings of this study are available from the corresponding author, [TK], upon reasonable request.
