# Supplementary material for: Reliability and structural validity of the Norwegian version of the TeamSTEPPS Teamwork Attitudes Questionnaire: A cross‐sectional study among Bachelor of Nursing students
Source: Nurs Open. 2020 Nov 4;8(2):664–74. doi: 10.1002/nop2.671 (PMC7877154; doi:10.1002/nop2.671)
Supplement: Supplementary file 1 — Supplementary Information [file NOP2-8-664-s001.pdf]

Spørreundersøkelse

Teamarbeid i helsetjenesten

Her er avklaringer av begrep som brukes i spørreundersøkelsen.

Et team kan defineres som en gruppe på to eller flere enkeltpersoner som er avhengig av hverandre i arbeidet mot et felles mål, og hvor det kreves samordning av innsats og ressurser for å oppnå et felles ønsket resultat og hvor alle deltagere har spesifikke roller eller funksjon. Team har ofte en tidsbegrenset deltagelse.

Teamarbeid i helsetjenesten beskrives som samspillet mellom to eller flere helsepersonell (team-medlemmer) som arbeider gjensidig avhengig av hverandre for å gi behandling og pleie til pasienter.

Teamledere er helsepersonell som har et forhåndsbestemt eller situasjonsbetinget ansvar for å lede og koordinere aktivitetene til andre gruppemedlemmer. (eks. vakthavende lege, visittansvarlig lege eller sykepleiere med gitt ansvar).

Behandlings og pleiepersonalet er helsepersonell som er involvert i den direkte pasientbehandlingen og pleien som en del av et tverrfaglig team (eks. leger, sykepleiere, hjelpepleiere, fysioterapeuter og ergoterapeuter).

Pasientsikkerhet defineres som «Vern mot unødig skade som følge av helsetjenestens ytelser eller mangel på ytelser»

En uønsket hendelse er en skade som er relatert til behandling og ikke til komplikasjoner ved et sykdomsforløp. Med behandling menes alle aspekter av helsetjenester inkludert diagnose, behandling, pleie, systemer og utstyr som brukes for å levere tjenestene.

Spørreskjema begynner på neste side. Husk å fylle ut bakgrunnsdata på siste side også.

Takk for at du tar deg tid til å svare på denne undersøkelsen.

Copyright Agency for Healthcare Research and Quality (AHRQ), USA 2008. Med tillatelse, oversatt til norsk av R. Ballangrud i samarbeid med M. L. Hall-Lord og O. Reiersdal, Seksjon sykepleie ved Høgskolen i Gjøvik 2015.

Teamarbeid i helsetjenesten

Vennligst svar på utsagnene nedenfor ved å krysse av (x) i boksen som stemmer overens med **din grad av enighet** – fra «Svært uenig» til «Svært enig». Vennligst velg kun ett svar for hvert spørsmål.

**Teamstruktur**		Svært uenig	Uenig	Nøytral	Enig	Svært enig
1	Det er viktig å be om tilbakemelding på behandling og pleie fra pasienter og deres pårørende.					
2	Pasienten er en viktig del av behandlings – og pleieteamet.					
3	Avdelingens ledelse har innflytelse på hvorvidt de som jobber i direkte pasientkontakt lykkes i arbeidet.					
4	Teamets oppdrag er viktigere enn de enkelte team-medlemmers individuelle mål.					
5	Dyktige team-medlemmer kan forutse hva de andre i teamet trenger av assistanse og hjelp i gjennomføring av oppgaver.					
6	Høyt spesialiserte team i helsetjenesten har mange fellestrekk med høyt spesialiserte team innen andre sektorer.					
**Ledelse**		Svært uenig	Uenig	Nøytral	Enig	Svært enig
7	Det er viktig at teamledere deler informasjon med team-medlemmene.					
8	Teamledere bør legge til rette for at team-medlemmer kan utveksle informasjon på en uformell måte.					
9	Dyktige teamledere ser på uønskede hendelser som en mulighet for å lære.					
10	Det er en teamleders ansvar å opptre som en god rollemodell når det gjelder teamadferd.					
11	Det er viktig at teamledere tar seg tid til å diskutere planen for hver enkelt pasient med de aktuelle team-medlemmene.					
12	Teamledere bør sørge for at team-medlemmene hjelper hverandre når det er nødvendig.					

Copyright Agency for Healthcare Research and Quality (AHRQ), USA 2008. Med tillatelse, oversatt til norsk av R. Ballangrud i samarbeid med M. L. Hall-Lord og O. Reiersdal, Seksjon sykepleie ved Høgskolen i Gjøvik 2015.

	Situasjonsovervåking	Svært uenig	Uenig	Nøytral	Enig	Svært enig
13	Alt personell kan bli opplært til å se etter viktige signaler i omgivelsene som kan ha betydning for pasientens situasjon.<br>(Med *Alt personell*, menes f.eks. renholdere, portører, bioingeniører og helsesekretærer)					
14	Overvåking av pasienter er viktig for å sikre et godt teamarbeid.					
15	Alt personell, også de som ikke er en del av helsepersonellteamet, bør oppfordres til å se etter og melde fra om endringer i pasientens tilstand.<br>(Med *Alt personell*, menes f.eks. renholdere, portører, bioingeniører og helsesekretærer)					
16	Det er viktig å være oppmerksom på de andre team-medlemmenes emosjonelle og fysiske tilstand.					
17	Det er riktig at et team-medlem tilbyr hjelp til en annen kollega som kan være for sliten eller for stresset til å utføre en oppgave.					
18	Team-medlemmer som er bevisste på sin emosjonelle og fysiske tilstand når de er på jobb, løser oppgavene sine bedre.					
	**Gjensidig støtte**	Svært uenig	Uenig	Nøytral	Enig	Svært enig
19	For å gjøre en god jobb bør team-medlemmene ha innsikt i arbeidet til de andre i teamet.					
20	Å spørre om hjelp er et uttrykk for at vedkommende ikke vet hvordan han/hun skal gjøre jobben sin på en god måte.					
21	Å hjelpe andre team-medlemmer, er et uttrykk for at den som hjelper ikke har nok å gjøre selv.					
22	Å tilby og hjelpe et annet team-medlem med hans/hennes arbeidsoppgaver, er en god måte å forbedre teamarbeidet på.					
23	Dersom du er bekymret for pasientsikkerheten, er det riktig å si tydelig fra, helt til du er sikker på at du har blitt hørt.					
24	Personlige konflikter mellom team-medlemmer påvirker ikke pasientsikkerheten.					

Fortsetter neste side, spørsmål 25-30

Copyright Agency for Healthcare Research and Quality (AHRQ), USA 2008. Med tillatelse, oversatt til norsk av R. Ballangrud i samarbeid med M. L. Hall-Lord og O. Reiersdal, Seksjon sykepleie ved Høgskolen i Gjøvik 2015.

Kommunikasjon		Svært uenig	Uenig	Nøytral	Enig	Svært enig
25	Det er betydelig større risiko for at det kan oppstå uønskede hendelser i team som ikke kommuniserer godt.					
26	Dårlig kommunikasjon er en av de vanligste årsakene til rapporterte uønskede hendelser.					
27	Uønskede hendelser kan reduseres gjennom god informasjonsutveksling med pasientene og deres pårørende.					
28	Jeg foretrekker å jobbe sammen med team-medlemmer som stiller spørsmål om informasjonen som jeg gir.					
29	Det er viktig å ha en standardisert metode for rapportering ved overlevering av pasient (eks. vaktskiftet, overflytting).					
30	Det er nesten umulig å lære personer hvordan de skal bli bedre til å kommunisere.					

Her kan du skrive kommentarer til svarene dine og til spørreskjemaet.

Copyright Agency for Healthcare Research and Quality (AHRQ), USA 2008. Med tillatelse, oversatt til norsk av R. Ballangrud i samarbeid med M. L. Hall-Lord og O. Reiersdal, Seksjon sykepleie ved Høgskolen i Gjøvik 2015.

Teamarbeid i helsetjenesten

Bakgrunnsdata

Vennligst svar på spørsmålene nedenfor ved å krysse av (x) i boksen som stemmer for deg.

1. Hva er ditt studiested?

Sett kun ett kryss

Trondheim ☐1
Gjøvik ☐2
Ålesund ☐3

☐ Jeg har deltatt i spørreundersøkelsen tidligere.

Har du deltatt i spørreundersøkelsen tidligere kan du avslutte her.

2.Har du tidligere utdanning fra universitet/høgskole?

Sett kun ett kryss

Ja ☐1
Nei ☐2

Hvis tidligere utdanning på universitet og høgskolenivå- angi hvilke type utdanning

3. Har du tidligere erfaring fra helsesektoren?

Sett kun ett kryss

Ja ☐1
Nei ☐2

4. Hvis tidligere erfaring fra helsesektoren. Hvor mange års erfaring har du?

Sett kun ett kryss

Mindre enn 1 år ☐1
1 -2 år ☐1
3-5 år ☐2
6-9 år ☐3
10-år eller mer ☐4

5. Kjønn

Sett kun ett kryss

Kvinne ☐1
Mann ☐2

6. Alder

Min alder er: …………………År.

Se gjerne over for å se om du har fått satt et kryss ved hvert spørsmål ☒

Takk for at du tok deg tid til å svare.

Skjemanummer.

Copyright Agency for Healthcare Research and Quality (AHRQ), USA 2008. Med tillatelse, oversatt til norsk av R. Ballangrud i samarbeid med M. L. Hall-Lord og O. Reiersdal, Seksjon sykepleie ved Høgskolen i Gjøvik 2015.
